# Recent Advances of the Polymer Micro/Nanofiber Fluorescence Waveguide

**DOI:** 10.3390/polym10101086

**Published:** 2018-09-30

**Authors:** Hongyan Xia, Tingkuo Chen, Chang Hu, Kang Xie

**Affiliations:** Dongyuan Synergy Innovation Institute for Modern Industries of GDUT, Guangdong University of Technology, Guangzhou 510006, China; hyxia@gdut.edu.cn (H.X.); chentingkuo@163.com (T.C.); huchang689566@163.com (C.H.)

**Keywords:** polymer micro/nanofiber, fluorescence waveguide, modulation, heterojunction, photonics components

## Abstract

Subwavelength optical micro/nanofibers have several advantages, such as compact optical wave field and large specific surface area, which make them widely used as basic building blocks in the field of micro-nano optical waveguide and photonic devices. Among them, polymer micro/nanofibers are among the first choices for constructing micro-nano photonic components and miniaturized integrated optical paths, as they have good mechanical properties and tunable photonic properties. At the same time, the structures of polymer chains, aggregated structures, and artificial microstructures all have unique effects on photons. These waveguided micro/nanofibers can be made up of not only luminescent conjugated polymers, but also nonluminous matrix polymers doped with luminescent dyes (organic and inorganic luminescent particles, etc.) due to the outstanding compatibility of polymers. This paper summarizes the recent progress of the light-propagated mechanism, novel design, controllable fabrication, optical modulation, high performance, and wide applications of the polymer micro/nanofiber fluorescence waveguide. The focus is on the methods for simplifying the preparation process and modulating the waveguided photon parameters. In addition, developing new polymer materials for optical transmission and improving transmission efficiency is discussed in detail. It is proposed that the multifunctional heterojunctions based on the arrangement and combination of polymer-waveguided micro/nanofibers would be an important trend toward the construction of more novel and complex photonic devices. It is of great significance to study and optimize the optical waveguide and photonic components of polymer micro/nanofibers for the development of intelligent optical chips and miniaturized integrated optical circuits.

## 1. Introduction to Polymer Optical Micro/Nanofibers 

Since the concept of “integrated optics” was first proposed by Dr. S.E. Miller of Bell Labs in the United States in 1969 [1], various types of micro-nano photonic materials and devices have been correspondingly studied and developed [2,3,4,5]. The great achievements of micro-nano-scale optical fibers supported the development of the internet industry and the rapid “shrinking” of the earth, and the basic theory of microfibers won the 2009 Nobel Prize. The high surface-to-volume ratios of one-dimensional optical micro/nanofibers that have tight optical confinement are fundamental to the construction of miniaturized photonics components and ultracompact optical circuits [6,7]. Against such a background, optical micro/nanofibers have been actively investigated in the international research field [8,9,10,11]. *Nature* has listed micro/nanofibers as one of the top five research hotspots in physics [12]. 

Optical micro/nanofibers usually refer to one-dimensional fibers or wires that are used for investigating light generation, transmission, detection, conversion, and modulation, with cross-sectional diameters that can be tuned from a few nanometers to a few micrometers (comparable to or less than the wavelength of light) and lengths spanning from hundreds of nanometers to millimeters. Micro/nanofibers made of glass have a long history and have been applied in signal transmission for many years due to their low transmission loss, but their mechanical properties are poor and they are not always compatible with other materials [13,14]. The other kind of micro/nanofibers that have been studied a lot are semiconductor optical micro/nanofibers, with a wide range of compositions (for example, Si, Ge, ZnO, CdS, GaN, GaAs, and InP), including group IV, II–VI, and III–V compounds and alloy structures [15]. The bandgap of semiconductor micro/nanofibers can be tuned from ultraviolet (UV) to near-infrared by changing their size and dimensions, offering an excellent platform for energy conversion in optic components [16]. The methods for fabricating semiconductor micro/nanofibers usually need harsh conditions (high-temperature and high-pressure environments), and high self-absorption energy loss and high fabrication costs are disadvantages [17]. Polymer optical micro/nanofibers composed of polymer matrix and other additives have only been developed in recent decades, and have attractive features compared to glass and semiconductor micro/nanofibers: (1) Simple modification: the chemical properties of polymer micro/nanofibers can be effectively tailored by simple surface modification, so the optical performance can be easily modulated using functional units [18]. (2) Easy doping: organic dye molecules, inorganic quantum dots, rare earth ion luminescent materials, metal nanostructures, enzymes, and even liquid materials can all be well-integrated into polymer optical waveguide systems at room temperature with high doping concentrations [19,20,21]. The diverse types of polymer optical micro/nanofibers can meet different applications, with a variety of light-emitting properties and optical functions. (3) Good flexibility: the “soft” characteristics of polymer matrix materials can greatly simplify the processing technology. Also, polymer composite functional systems can be obtained by various methods and are compatible with traditional semiconductor and glass materials, which is advantageous for large-scale production at low cost [22,23]. A series of characteristics and advantages of polymer materials, micro-nano structures, and optical principles are incorporated into polymer optical micro/nanofibers that can better transmit and regulate photon signals; this is one of the leading research directions in the field of micro-nano photonics for future development [24,25]. Although the past decades have seen tremendous progress in the field of polymer optical micro/nanofibers, many limitations and challenges still remain. Reducing optical loss of polymer micro/nanofibers is always desirable, which is the primary issue in the development of polymer optical fibers. In order to better meet actual application requirements, the other issue is improving thermal and chemical stability, on which much research should be conducted further down the road.

The earliest polymer optical fiber can be traced back to 1980 and the DuPont Company in the United States, which developed one kind of optical fiber whose core material was polymethyl methacrylate [26]. Polymer optical fibers have the advantages of good flexibility, easy processing, high coupling efficiency, low price, light weight, etc., which make them suitable for local area networks with short-distance multipoint access [27]. Today, polymer micro/nanofibers are used to make optical devices (such as micro/nanoscale waveguides, photodetectors, and optical sensors) and are expected to be a promising candidate for light processing in future micro/nanophotonic fields [28,29,30,31]. 

## 2. Polymer Micro/Nanofiber Fluorescence Waveguides

**Figure 1:**
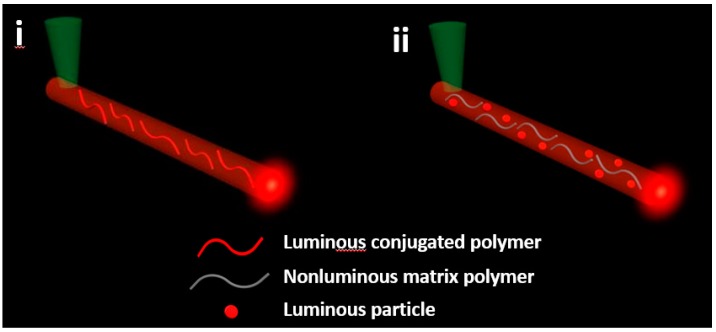
Structure of luminescent polymer micro/nanofibers: (i) the micro/nanofiber is made of self-luminous conjugated polymers; (ii) the micro/nanofiber is composed of nonluminous matrix polymers and doped luminescent dyes (organic and inorganic luminescent particles).

The polymer micro/nanofiber waveguide in this paper refers to the fluorescence waveguide rather than the laser waveguide; that is to say, the propagated light is from the photoluminescence (PL) of the polymer micro/nanofiber itself upon optical excitation, but not the excited laser. According to the nature of luminescence, polymer luminescent micro/nanofibers can be either intrinsic or loaded. The most typical example of intrinsic polymer luminescence micro/nanofibers is those composed of conjugated polymers (such as polyfluorene, polyacetylene, poly (p-phenylene), etc.). Conjugated polymers are a kind of polymer with a long-range π-electron conjugation; there are energy bands between the bonding and antibonding orbits of the π-electron that lead to their luminescent characteristic. Before the appearance of intrinsic luminescent polymers, various luminescent materials were loaded into the polymers by doping or copolymerization to form various kinds of polymer luminescent micro/nanofibers. The loaded method not only makes full use of the advantages of high optical transparency, easy processing, and low cost of the polymer system, but also maintains the excellent optical properties of luminescent materials, which are widely used in the preparation of various fluorescent materials. The composition of these two forms of light-emitting polymer micro/nanofibers is shown in Figure 1. The micro/nanofibers emit PL under the excitation of excited light, and the PL at the excitation point can be propagated along the entirety of the micro/nanofibers; then, the fluorescence signal at the end can be collected and analyzed to study the influence of different parameters (such as the micro/nanofibers’ morphology and the different structures, contents, and emission colors of the doped light-emitting molecules) on the generation and transmission of light signals. After understanding the process and mechanism, the applications of optical waveguides of polymer micro/nanofibers in the construction of various types of micro-nano optics chips and photonics components can be explored, and finally, applied to integrated optical circuits.

**Figure 2:**
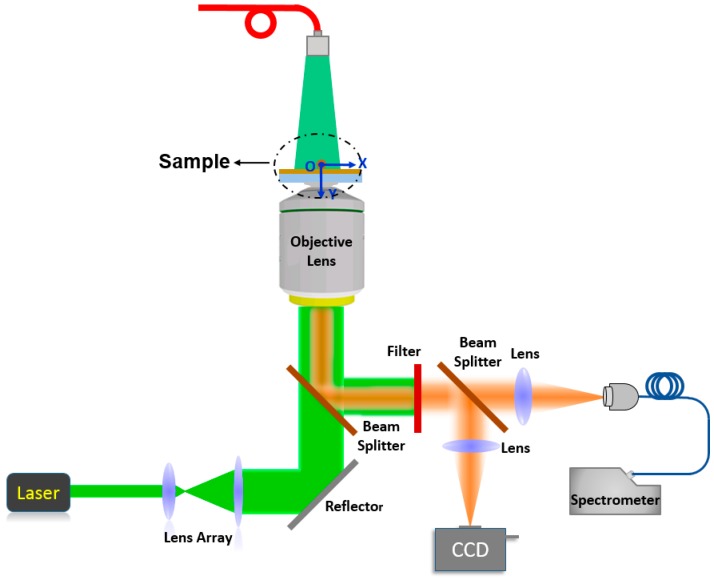
Experimental setup for single-fiber optical waveguide experiments (CCD here is shorted for Charge-coupled Device).

Optical waveguiding properties and the outcoupled tip fluorescence emissions of polymer micro/nanofibers were studied by a single-fiber fluorescence imaging system (Figure 2). Here, the guided light is from the intrinsic fluorescence of conjugated polymers or the doped luminous particles, so this fluorescence waveguide is classified as an active waveguide. A focused laser beam is focused on the micro/nanofibers by an oil-immersed objective to excite the interior fluorescence. The emission of the micro/nanofibers is then collected by the same objective. A filter here is used to reject the incident laser beam and allow the fluorescence of micro/nanofibers to reach the detector. A beam splitter here is used to split the fluorescence into two beams: the first beam is applied to collect fluorescence images, and the other beam is applied to record the tip emission spectra through a spectrometer. The additional laser on the top side of the objective is to evenly illuminate all areas of the long fluorescent micro/nanofibers.

### 2.1. Fabrication of Polymer Optical Micro/Nanofibers

The preparation methods of polymer micro/nanofibers with the fluorescence waveguide generally include the template method [32], direct stretching method [33], self-assembly method [34], and electrospinning method [35]. Research teams at home and abroad, such as Redmond [36], Tong [37], Zou [38], and Pisignano [39], among others, have done many studies on polymer optical micro/nanofibers and the related photonics components.

**Figure 3:**
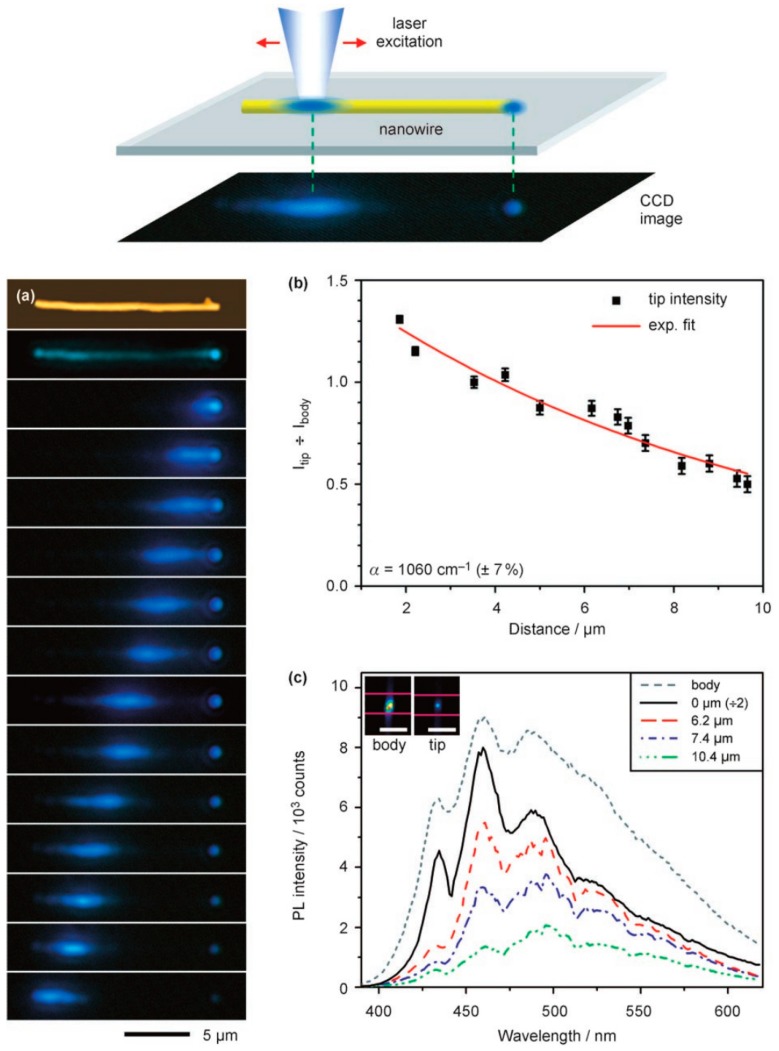
Fluorescence waveguide properties of the prepared poly(9,9-dioctylfluorene) (PFO) nanofiber. Top panel: Schematic diagram of the single nanofiber fluorescence waveguide setup. (**a**) Atomic force microscopy (top image) and fluorescence microscopy (second image from top) images. Fluorescence microscopy images obtained as a laser excitation spot at different positions along the same PFO nanofiber (bottom 13 images). (**b**) Data of *I*_tip_/*I*_body_ versus different propagation distances and the exponential fit for the fluorescence waveguide (*I* is shorted for the fluorescence intensity). (**c**) Photoluminescence (PL) spectra of outcoupled emission from the tip of the PFO nanofiber for different propagation distances (the whole Figure 3 was reprinted with permission from O’Carroll et al. [40]).

Generally, preparation of polymer micro/nanofibers from the template method is as follows: First, the polymer melt is injected into the porous template by means of vacuum annealing, and then micro/nanofibers are formed within the template pores by controlling the cooling process, and selective dissolution of the outer template will obtain the polymer micro/nanofibers. In 2007, Redmond et al. synthesized poly(9,9-dioctylfluorene) (PFO) polymer nanofibers using a porous alumina template method [40]. The prepared PFO nanofibers exhibited characteristic blue PL upon optical excitation, and the locally excited fluorescence could be propagated along the nanofibers and outcoupled from the tips, as shown in Figure 3a, suggesting that the obtained PFO nanofibers behaved as active fluorescence waveguides. By recording the change of the outcoupled waveguided fluorescence spectra at the tips of the PFO fibers with different propagation distance (from the excited spot to the outcoupled tip, Figure 3c), the propagation loss was calculated (0.48 dB µm^−1^, as shown in Figure 3b). 

**Figure 4:**
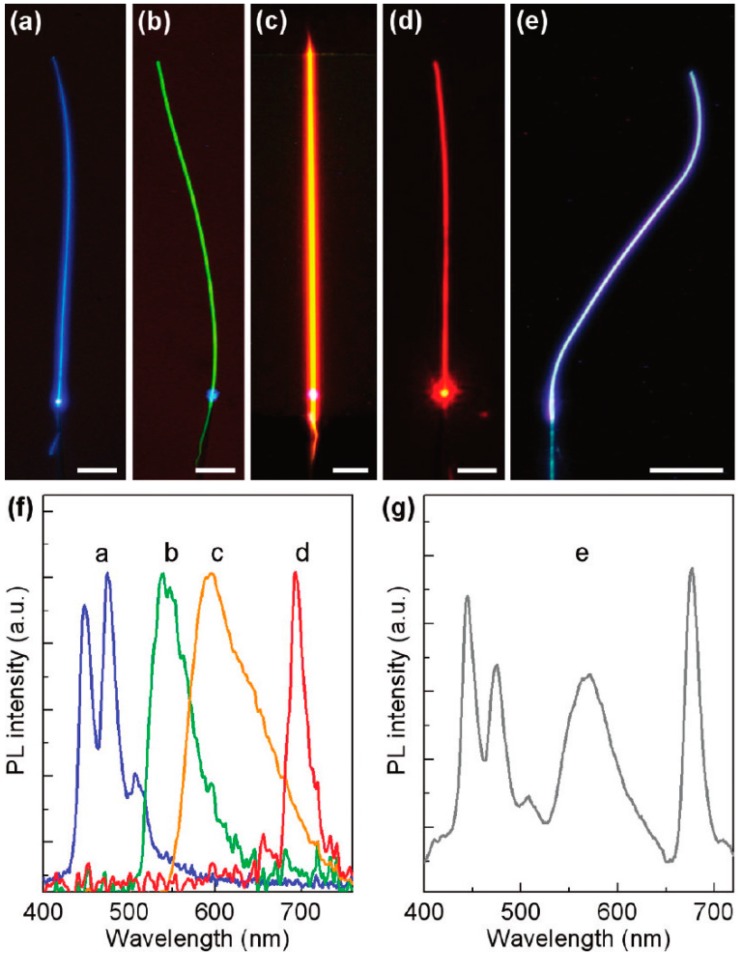
(**a**–**e**) Fluorescence microscopy images of a nanofiber with blue, green, orange, red, and white fluorescence emission, respectively. (**f**,**g**) PL spectra corresponding to the nanofiber emissions shown in a–e (the whole Figure 4 was reprinted with permission from Gu et al. [41]).

Direct drawing is a general and simple approach to obtain light-emitting polymer micro/nanofibers. Polymer solution with doped dyes is stirred at room temperature to form a uniform mixture. A needle with a sharp tip is often used to transfer a drop of mixture solution onto a thin piece of glass substrate and then draw a fiber from the droplet quickly, and after the solvent evaporates, polymer micro/nanofibers form on the glass substrate. In 2010 and 2011, various organic (rhodamine, perylene, fluorescein, etc.) and inorganic (quantum dot) light-emitting molecules were doped into polymer solutions by Tong’s group, and a series of fluorescence waveguides based on polymer micro/nanofibers were prepared by directly drawing from polymer solutions with dissolved fluorescent dyes [41,42]. Upon laser excitation from one end, different colors of bright fluorescent emissions were generated and propagated along the micro/nanofibers (Figure 4a–e). The fabricated polymer micro/nanofibers incorporated with various fluorescent dyes generated emissions of desired colors that covered the entire visible spectrum (Figure 4f). By simultaneously doping with multiple fluorescent dyes, optical waveguide micro/nanofibers with white light can be obtained (Figure 4g), which is expected to be applied to the preparation of micro/nanoscale white light sources. 

**Figure 5:**
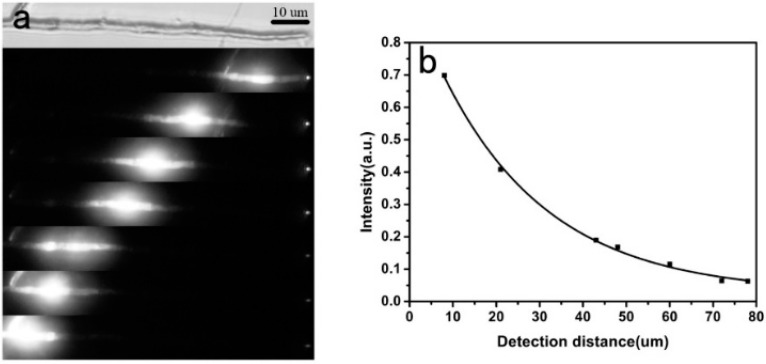
(**a**) Fluorescence microscopy images collected upon different excitation positions of the same microtube. (**b**) *I*_tip_/*I*_body_ versus propagation distance and the exponential fit (*I*_tip_ and *I*_body_ are shorted for the fluorescence intensity from the tip and the body of the microtube, respectively, the whole Figure 5 was reprinted with permission from Hu et al. [43]).

Zou et al. fabricated one-dimensional polydiacetylene (PDA) hollow microtubes by hierarchical assembly under certain conditions in 2013 [43]. First, mixtures of the monomers melamine-substituted diacetylene and diacetylene were dissolved in a minimum amount of ethanol. Then, the ethanol solution was injected into 300 mL buffered aqueous solution and sonicated at 70 °C for 90 min. After the solution was cooled and stored at 4 °C in a refrigerator overnight, they obtained diacetylene compound vesicles. They dispersed the 300 mL vesicle solution in 30 clean small bottles (each containing 10 mL solution) and added 10 µL of Pb^2+^ solution (concentration 10^−3^ M) to each bottle and placed them in a ventilated place at room temperature (25 °C), and white filaments precipitated after two weeks, which were the assembled microtubes. They observed the continuous morphologies to study the mechanism of microtube formation and found that the initial diacetylene vesicles formed fibers under the coordination of Pb^2+^, then different fibers combined with each other to form a wide fiber layer structure. After about 10 days, the surfaces stacked on top of each other to form layered structures with a certain thickness. Finally, the layered structures curled to spontaneously form hollow microtubes. After UV light irradiation and heating, the diacetylene monomer polymerized into polymer PDA and transformed from the colorless nonfluorescent blue phase to the fluorescent red phase. When excited at the middle of the microtube, the fluorescence of the red-phase PDA could be propagated along the microtube and emitted at the end, indicating characteristic waveguiding behavior (Figure 5); thus, they realized the optical waveguide in a polymer PDA microtube for the first time. 

**Figure 6:**
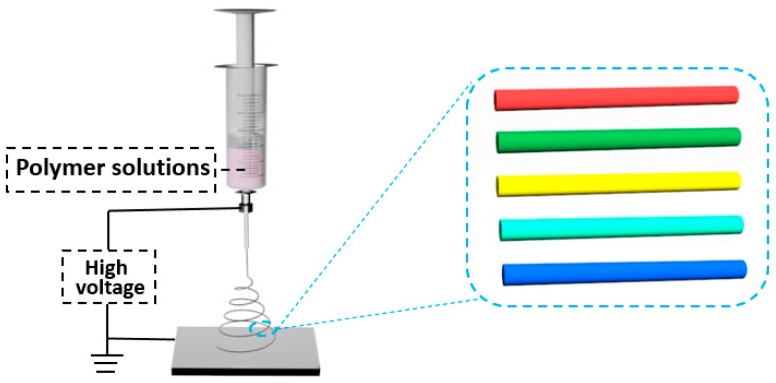
Schematic of electrospinning process for polymer micro/nanofibers.

Electrospinning is cost-effective and versatile for polymer micro/nanofiber fabrication, in which a polymer solution or melt is spun under a strong electric field, and the droplet at the needle changes from a spherical to a conical shape (Taylor cone) and extends from the conical tip to obtain fiber filaments (Figure 6) [44,45]. The morphology and diameter of micro/nanofibers can be modulated by different spinning parameters (such as solution concentration, voltage, temperature, and humidity of the environment, needle shape, and distance between the needle and the receiving screen). Electrospinning enhances the polymer chain alignment and polarization along the fiber axis, leading to significant enhancement of the emission quantum yield. Pisignano et al. did a lot of work on electrospun polymer optical micro/nanofibers in 2013 and 2015 and proposed many solutions for how to improve the performance and reduce the optical transmission loss [46,47]. They extended the polymer micro/nanofiber emission from the visible and near-infrared light range to the ultraviolet light range, making the fibers interesting for building light-emitting devices. They also obtained smoother and more uniform micro/nanofibers with enhanced optical properties for the first time by electrospinning in a controlled nitrogen atmosphere instead of the conventional process performed in air. Moreover, by adding organic salts to the electrospinning solution, ultrafine polymer nanofiber optical waveguides can be realized. These findings help the polymer micro/nanofibers be better used as optically active elements for photonics and light-emitting optoelectronic devices.

### 2.2. Low-Transmission Loss of Polymer Micro/Nanofiber Waveguides 

**Figure 7:**
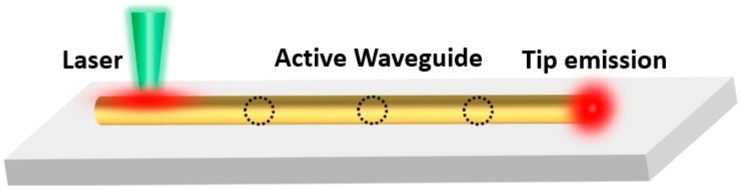
Scheme of waveguide loss coefficient measurement.

As shown in Figure 7, for the same polymer micro/nanofiber, we changed the position of the excitation point, and recorded the fluorescence intensity of the excitation point and tip emission point and the distance between the tip and the location of optical excitation to study whether the relationship between the waveguided light intensity and the light transmission distance would obtain the loss coefficient of the optical waveguide. The calculation formula is *I*_tip_/*I*_body_ = e^−αx^, where α is the propagation loss coefficient, *I*_tip_ is the fluorescence intensity measured at the tip, *I*_body_ is the body fluorescence intensity at the excitation spot, and x is the propagation distance [48]. The main direction in developing optical waveguides based on polymer micro/nanofiber systems is how to reduce the optical transmission loss, since further reduction of transmission loss is the primary problem, to better meet the needs for the application of optical communication and photonics components. 

The main waveguide transmission losses of polymer optical micro/nanofibers are self-absorption of the materials, light scattering caused by the irregular internal structure, and energy dissipation into the substrate (glass or silicon wafer) [49]. The following formula requirement must be met if light can be guided within the polymer micro/nanofibers: m < (2d/λ)(*n*_ew_^2^ − *n*_0_)^0.5^, where m is the light transmission mode supported by the micro/nanofibers, λ is the wavelength of the waveguided light, and *n*_ew_ and *n*_0_ are the refractive indices of the micro/nanofibers and air, respectively. The value of m must be at least 1 if the light signals can be transported [40], so the polymer micro/nanofibers cannot transport optical signals if the fiber radius is too small (for example, a nanofiber will not transport a light signal with a wavelength of 632.8 nm when the radius is less than 125 nm). This is because the light signals in the ultrafine nanofibers are easily leaked into the glass or silicon substrates and thus cannot form the waveguide effect. In 2007, Redmond et al. fabricated polyfluorene nanofibers with a diameter of approximately 300 nm by alumina templates. These nanofibers exhibited significant fluorescence waveguide behavior under excitation light. By calculation based on the above formula, they found that when the diameter of nanofiber is less than 150 nm, fluorescence of polyfluorene (wavelength 460 nm) will not be propagated [40]. The experimental results showed the same results, which agree well with the theoretical calculation. 

**Figure 8:**
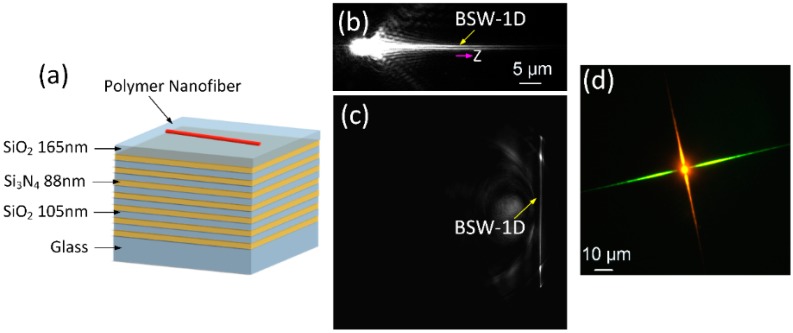
Optical waveguide of ultrafine polymer nanofibers on the surface of a multilayer dielectric film. (**a**) Diagram of the structure. (**b**) Front focal plane image of optical signal propagating along the polymer nanofiber. (**c**) Back focal plane image of optical signal propagating along the polymer nanofiber. (**d**) Fluorescence image of two crossed nanofibers that propagated light signals with different wavelengths ( Here the BSW is shorted for the Bloch Surface Wave, the whole Figure 8 was reprinted with permission from Wang et al. [50]).

In order to solve the above problem, Zou et al. placed ultrafine polymer nanofibers (diameter 120 nm) on the surface of a multilayer dielectric film (top layer is SiO_2_, a conventional glass material) to block the leakage of optical signals by means of the photonic bandgap of the multilayer film [50]. As shown in Figure 8a–c, optical signals can be transmitted in ultrafine polymer nanofibers. Moreover, they changed the polymer nanofiber waveguiding properties by doping. Figure 8d demonstrates the luminescent particles in different polymer nanofibers combined on the same substrate, where nanometer optical waveguides with different emission wavelengths were successfully realized, which has not been achieved using conventional processing methods to date. This work provides a solution for how to prevent light signals in ultrafine nanofibers from leaking into the substrates and lays the foundation for practical applications of ultrafine polymer nanofiber optical waveguides.

**Figure 9:**
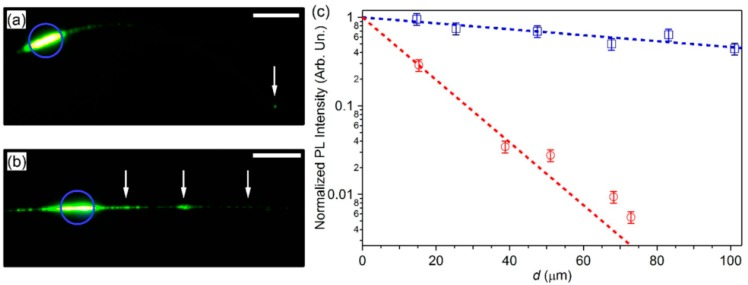
Waveguiding micrographs of polymer spun fibers in (**a**) controlled atmosphere and (**b**) air, and excited by a focused laser (blue circles). Scale bars = 20 μm. White arrows in (**a**,**b**) show the fiber tip and defects along the fiber axis, respectively. (**c**) Waveguided PL intensity along a single fiber versus propagation distance d, with fibers electrospun in controlled atmosphere (blue squares) and air (red circles). Dashed lines: exponential fit to decay (the whole Figure 9 was reprinted with permission from Fasano et al. [47]).

Pisignano et al. explored many methods to reduce the optical transmission loss of polymer micro/nanofibers. They found that the surface of light-emitting conjugated polymer (poly-p-phenylene vinylene) micro/nanofibers obtained by electrospinning under the protection of a controlled gas flow (nitrogen atmosphere) were smoother than those obtained by directly spinning in air [47]. The reason was the decrease of moisture and oxygen interference in the ejected jet, leading to decreased surface defects, resulting in light scattering and optical transmission loss reduction. Figure 9 demonstrates the differences in light propagation loss for polymer fibers spun in the controlled atmosphere and air. We can see that there are many obvious defects on the fiber surface in Figure 9b compared with Figure 9a, causing light to leak out. Optical loss coefficient for fibers electrospun in nitrogen was about 80 cm^−1^, which was much lower than that for fibers electrospun in air (800 cm^−1^), as determined through calculation (Figure 9c). The above experimental results indicate that the controlled atmosphere can prevent defect production and decrease light scattering, leading to a great reduction (in orders of magnitude) in optical transmission loss and improvement of optical waveguide performance.

**Figure 10:**
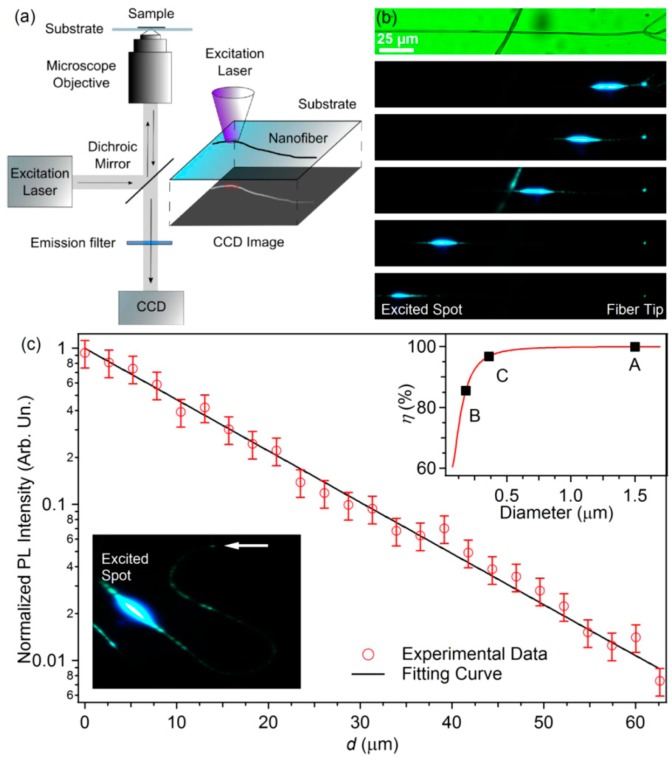
(**a**) Scheme of single-fiber waveguiding characterization setup. (**b**) Images of a fiber excited by a focused laser at different positions along the fiber. Top panel: brightfield image of the nanofiber. (**c**) Fluorescence intensity (red circles) versus guided distance d from the excitation spot to the fiber tip and the exponential fit. Bottom-left inset: micrograph of light guided in a bent polymer fiber. Right-top inset: fraction of guided power in fundamental mode versus fiber diameter. Points labeled A, B, and C correspond to the average diameter of fibers fabricated without additives (A) and with the addition of organic salts (B and C) (the whole Figure 10 was reprinted with permission from Fasano et al. [46]).

Many polymer micro/nanofibers electrospun from a single solvent have beaded structures and large diameters. The polymer is not always easily dissolved due to the large molecular weight; a good solvent can prevent aggregation and make the polymer molecules better aligned and oriented. However, good solvents for many polymers often have a low boiling point and poor conductivity, which goes against the electrospinning process. Organic salts and other additives can increase the conductivity without affecting the viscosity and surface tension of the solution and are often used to improve the electrospun fiber waveguiding properties. Pisignano et al. demonstrated that fibers obtained from fluorene-substituted phenylenediamine chloroform solution, to which was added organic salt (tetrabutylammonium iodide), were more regular than those obtained using a single solvent (chloroform); also, the sizes were more uniform and nanofibers with very fine diameters (<10 nm) could be obtained [46]. Figure 10 shows fibers with an optical loss coefficient of about 100 cm^–1^ electrospun from the salt mixture solution, while higher loss coefficient values were measured for fibers obtained from polymer solutions without adding salt. These experiments and discoveries provide an important theoretical basis for how to prepare polymer micro/nanofibers with different functions and improve their optical waveguide properties.

**Figure 11:**
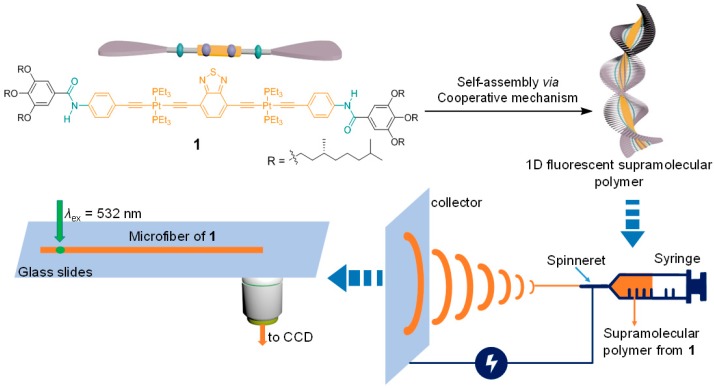
Diagram of “nucleation-chain growth” synergistic supramolecular polymerization system and its optical waveguide application (ex is shorted for the excitation light, λ_ex_ is referred to the wavelength of the excitation light, the whole Figure 11 was reprinted with permission from Wang et al. [51]).

As we know, problems exist, such as nonuniform sizes and amorphous structures for π-conjugated polymer optical waveguides, so Zou et al. proposed a new strategy for controlled supramolecular polymerization of π-conjugated monomer molecules and obtained supramolecular polymer microfibers with excellent optical waveguide behavior by bionic self-assembly and electrospinning in 2017 [51]. The constructed supramolecular polymerization system can effectively avoid the defects and achieve optimization of optical waveguide performance. Through rational molecular design of π-conjugated platinum alkyne monomers, with the help of efficient addition of noncovalent driving forces (hydrogen bonding and π–π stacking), they obtained a one-dimensional supramolecular conjugated polymer based on the nucleation-chain-growth synergistic polymerization mechanism. It was confirmed that the prepared supramolecular polymer had a high degree of polymerization, solution processability, and excellent luminescent properties, as determined by the combination of spectroscopic methods and mathematical models (Figure 11). Compared with the traditional π-conjugated polymer systems, light transmission loss of the prepared polymer microfiber waveguide showed a significant decrease (calculated optical loss coefficient was 18.9 cm^−1^, two orders of magnitude below the reported optical loss coefficient for π-conjugated systems). This provides a new method for the research of π-conjugated polymer optical waveguides. 

### 2.3. Modulation of Polymer Micro/Nanofiber Waveguides 

Optical modulation of waveguides and logic gates has important applications in highly integrated optical communication devices, optical computing, and photonic circuits [52,53]. Although there are extensive studies on the waveguide properties of active polymer micro/nanofibers, the flow of light within them is always predetermined after fabrication and cannot be readily modulated during operation, limiting their practical application in optical communication components and integrated optoelectronic devices. Thus, developing novel optical modulation means of waveguiding in polymer micro/nanofibers should be an interesting and challenging task for future information technology. 

**Figure 12:**
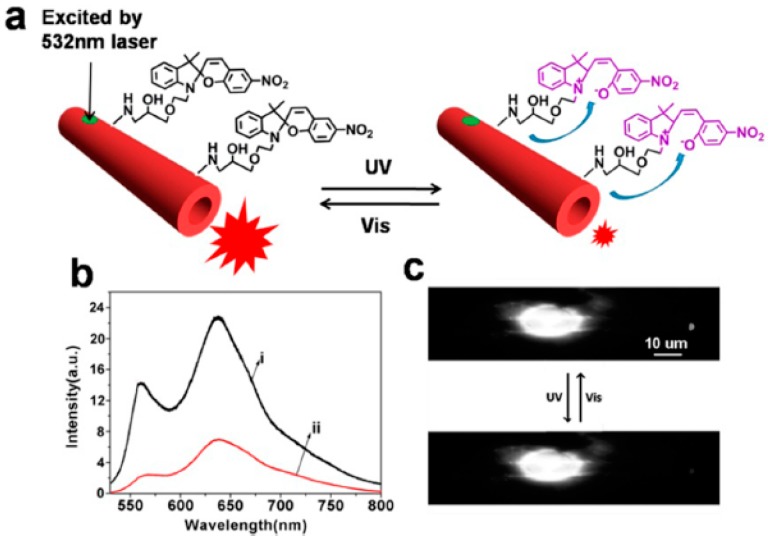
(**a**) Modulation of waveguiding in a PDA microtube. (**b**) Tip emission spectra of the microtube after irradiation with visible light and UV light. (**c**) Fluorescence microscopy images collected upon excitation of the same microtube following UV and visible light irradiation (the whole Figure 12 was reprinted with permission from Xia et al. [56]).

The polymer PDA, which has a long conjugated backbone, will change its effective conjugation length under external stimuli (such as temperature, pH, ion, pressure, solvent, coordination, biological interaction, etc.), conforming to a change of the main chains, resulting in a blue to red color transition accompanied by the appearance of fluorescence [54]. These color and fluorescence changes provide a broad platform for the preparation and study of various optical modulators, chemical biosensors, and optoelectronics devices, because they are controllable [55]. Zou et al. functionalized a PDA microtube with photoresponsive spiropyran by surface chemical modification and constructed a new type of photomodulation waveguide based on fluorescence resonance energy transfer (FRET) [56]. As shown in Figure 12, under UV light irradiation (365 nm), spiropyran was isomerized from the ring-closed form to the ring-opened form and could effectively quench the fluorescence and waveguiding of the microtube due to the intense absorption (in the range of 500 nm to 600 nm). After visible light irradiation (435 nm), the ring-opened spiropyran isomerized back to the ring-closed form, which has no absorption, and the fluorescence and light waveguiding of the microtube returned to the original value. The above optical modulation process can be repeated many times without obvious energy degradation due to the good photoisomerization property of spiropyran. Photomodulation of polymer microtube waveguides is carried out through external light stimuli. 

**Figure 13:**
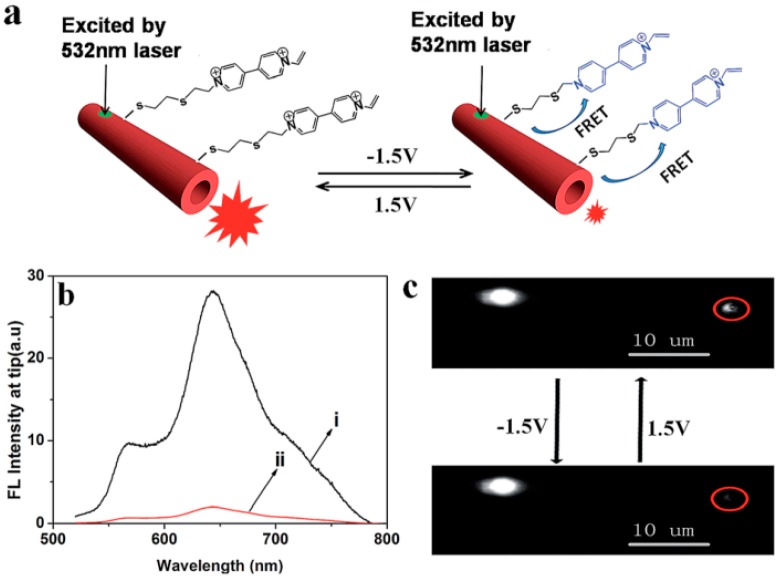
(**a**) Electrical modulation of waveguiding in the PDA microtube. (**b**) Tip emission spectra of the microtube with applied voltage of 1.5 V and −1.5 V. (**c**) Fluorescence microscopy images collected upon excitation of the same microtube following driven voltage of 1.5 V and −1.5 V (the whole Figure 13 was reprinted with permission from Yang et al. [57]).

By the same principle, they successfully fabricated a viologen-functionalized PDA microtube, with waveguiding emission that could be modulated by external electrical stimuli [57]. When applying a voltage of −1.5 V to the microtube, the colorless viologen that was in the MV^2+^ state turned to the blue MV^+^ state, whose absorption spectra overlap with the fluorescence of the red-phase PDA, leading to a dramatic decrease of microtube fluorescence and waveguiding emission due to the FRET (fluorescence resonance energy transfer) mechanism. However, when applying a voltage of +1.5 V to the microtube, the MV^+^ viologen on the surface isomerized back to the colorless MV^2+^ state and the fluorescence intensity and waveguiding emission at the tips returned to their original values, because there was no FRET between the MV^2+^ viologen and the PDA (Figure 13). Modulation of waveguide intensity in polymer microtubes upon electrical stimulus has also been realized successfully. Also, they constructed a series of “OR” and “INHIBIT” logic gate operations based on the above modulation of the polymer micro/nanofiber waveguide process, providing a theoretical basis and key technologies for the preparation of new intelligent photonics devices.

**Figure 14:**
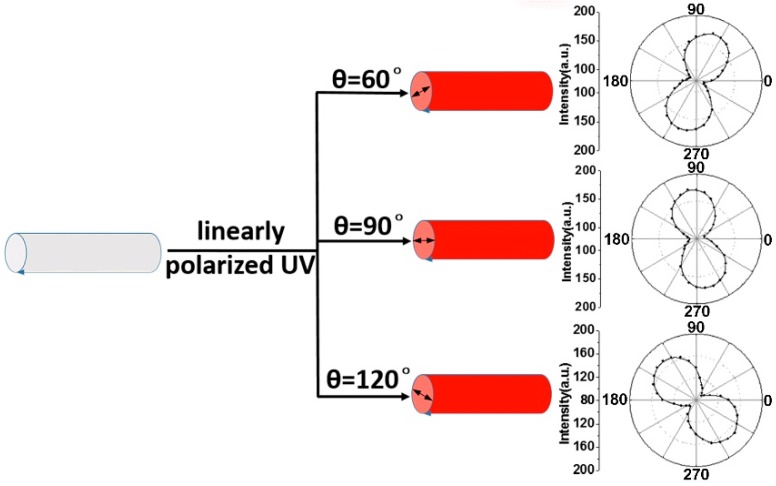
Modulation of waveguide polarization for optical polymer microfibers (the whole Figure 14 was reprinted with permission from Xia et al. [58]).

Aside from optical waveguide intensity, Zou et al. modulated the waveguide polarization of polymer microfibers by controlling the arrangement of polymer chains within the fibers [58]. They synthesized polystyrene (PS) microfibers doped with diacetylene (a mixture of chloroform solution with 5 wt % diacetylene and 20 wt % PS) using the electrospinning method. After polymerization and heating, the monomer diacetylene polymerized into polymer PDA, microfibers embedded with the PDA were fluorescent, and the emitted fluorescence from the interior of the red-phase PDA could be guided along the microfibers as a characteristic waveguide. During the process of the monomers evolving into polymers, the predominant orientation of the PDA main chains inside the microfiber can be optically controlled by tuning the polarization direction of the UV light, and then the polarization states of the optical waveguided fluorescence will be subsequently modulated (Figure 14). The polarization-adjustable waveguide polymer microfibers can be applied for the construction of smart polarization-related photonic components.

### 2.4. Application of the Polymer Micro/Nanofiber Fluorescence Waveguide

Due to its excellent photonic properties, flexible manufacturing process, low cost, good compatibility with substrates, and low-dimensional advantages, the polymer micro/nanofiber fluorescence waveguide has widespread applications in micro/nanometer-scale waveguides, light-emitting devices, photodetectors, and optical sensors [59,60,61]. With ever-growing demand and ongoing research, polymer optical micro/nanofibers will continue to play more and bigger roles in future integrated photonic applications. Here, we will present several examples of applications of the polymer micro/nanofiber fluorescence waveguide. 

**Figure 15:**
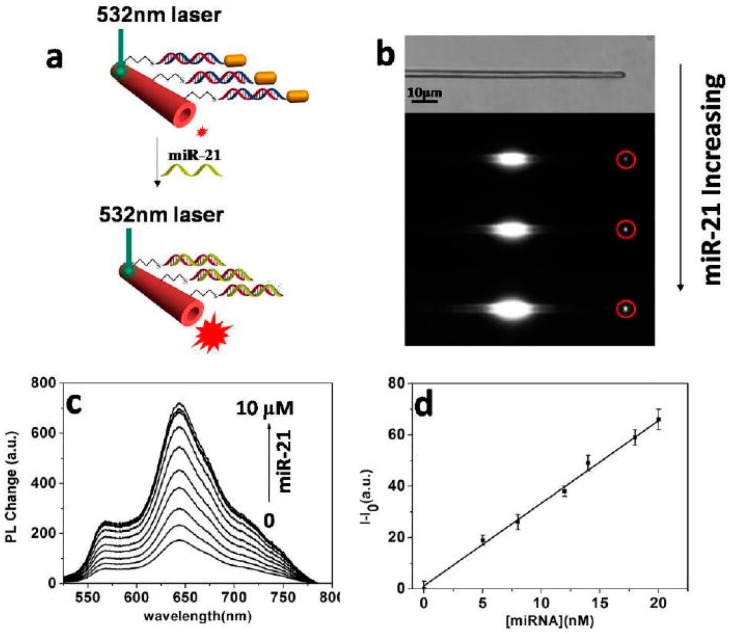
Detection of miRNA-21 based on Au@PDA microtube waveguide. (**a**) Schematic diagram. (**b**) Microscopy of PDA microtube and Au@PDA microtube waveguide sensor with increasing miR-21. (**c**) PL changes for tip emission of PDA microtube upon increasing miRNA-21. (**d**) Plot of PL changes of the waveguided tip emission at 640 nm upon addition of miR-21. *I*_0_ and *I* represent PL intensity before and after reaction with miRNA-21, respectively (the whole Figure 15 was reprinted with permission from Zhu et al. [64]).

In recent years, the detection of MicroRNAs (miRNAs, as a kind of endogenous, short, non-coding single-stranded RNA molecules) has become a hot topic in complex biological environments, with the development of medical technology. miRNAs are now proven to serve as tumor markers for early cancer detection, therefore it is of great significance to find a simple, fast, efficient, and sensitive method to detect miRNAs [62]. Early miRNA detection methods could not be made into integrated sensors, which limited their practical application. More importantly, detection of miRNAs requires cumbersome sample preparation and amplification steps. Recent research has focused on electrochemical methods, fluorescence methods, electrochemiluminescence, colorimetric analysis, signal amplification, and so on [63]. Zou et al. prepared gold nanorod-modified PDA microtubes (Au@PDA microtubes) to realize highly sensitive detection of miRNA-21 (Figure 15a) [64]. The PDA microtube has one-dimensional optical waveguide characteristics, and the excitation and emission positions of the fluorescence are separated from each other, which helps to reduce the interference of background fluorescence in the biological sample. The intensity of waveguided PL at the tip of the PDA microtube was used to indicate the detection process (Figure 15b). At the same time, they designed the chain replacement reaction triggered by the toehold to make detection more specific. The detection limit was reduced to 0.01 nM based on the concentration-enrichment effect (Figure 15c,d), which is equivalent to the concentration of miRNAs in the serum of cancer patients, and they successfully applied this method to miRNA-21 detection in human serum. The PDA microtube optical waveguide detection system can be further designed as a portable medical diagnostic biochip for real-time monitoring. 

**Figure 16:**
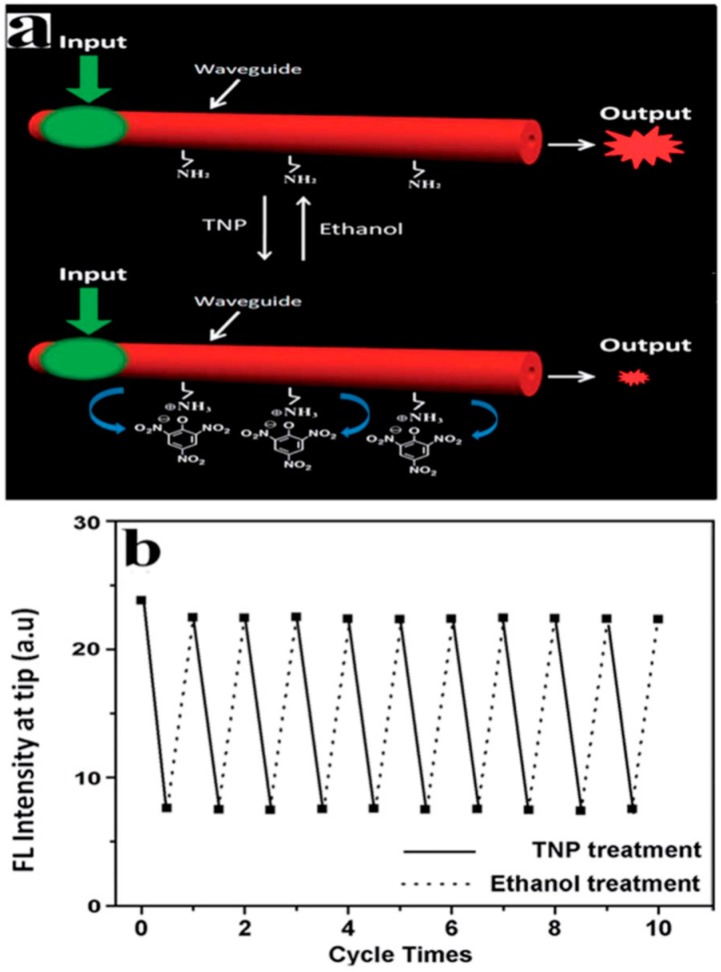
Detection of trinitrophenol (TNP) based on optical response of a PDA-microtube waveguide sensor. (**a**) Schematic diagram. (**b**) Changes of the outcoupled tip emission intensity (at 640 nm) of the PDA microtube waveguide sensor upon repeated TNP sensing and regeneration cycles (FL in the figure is shorted for the fluorescence, the whole Figure 16 was reprinted with permission from Yang et al. [67]).

Trinitrophenol (TNP) is a nitro explosive that is biologically toxic and has been used in dyes, pharmaceuticals, chemical laboratories, and many other settings. There are many hidden dangers in TNP that can cause pollution to the soil and groundwater [65]. Most of the current tests rely on complicated instruments and equipment, and quickly and effectively detecting TNP is particularly important [66]. Zou et al. used the one-dimensional polymer PDA microtube as a carrier for TNP detection, because the surface of the microtube is positively charged due to the presence of the amino group, while the surface of the TNP is negatively charged, thus there is electrostatic adsorption between the microtube and TNP, causing the fluorescence of PDA to transfer to TNP, leading to the fluorescence quenching and decreased waveguide intensity, accordingly. Experiments have shown that PDA microtubes show specific recognition of TNP for the same kind of nitro compounds, and the waveguide intensity of the end emission and the concentration of TNP show a good linear relationship in the low-concentration ranges, which can be used in the specific detection of explosive TNP. After the TNP attached to the surface of the microtube was washed with ethanol, the waveguide of the microtube recovered to the original intensity. The above detection process could be repeated many times without affecting the waveguiding properties of the PDA microtube (Figure 16). With the above experimental results, they realized the detection of TNP based on the PDA microtube, which was used as a reversible fluorescent probe [67]. The utility of a single PDA microtube as a TNP-detecting sensor is that it exhibits a rapid response and high sensitivity, and it is expected to be integrated on microchips for remote detection in complex chemical and biological environments.

**Figure 17:**
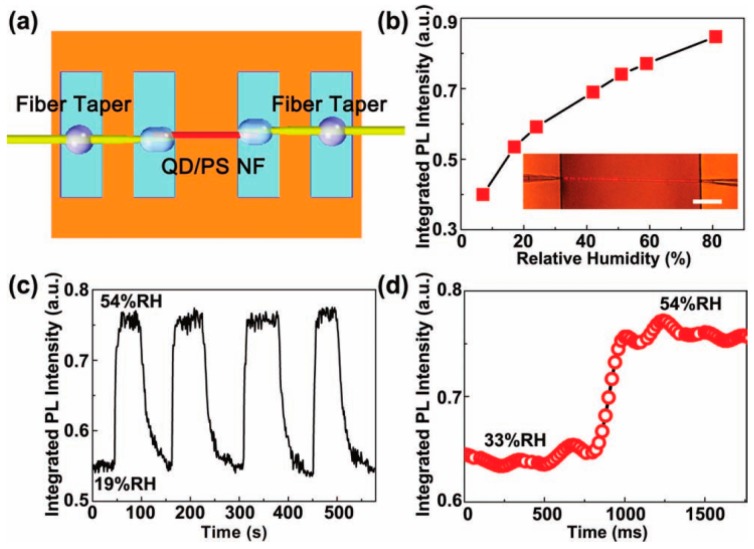
Quantum dot (QD)/polystyrene (PS) single-nanofiber humidity sensor. (**a**) Schematic diagram. (**b**) Integrated PL intensity of the polymer nanofiber exposed to relative humidity (RH) from 7% to 81%. Inset: microscopy image of the polymer nanofiber upon light excitation used in the sensor. (**c**) Response of the nanofiber sensor to alternately changed relative humidity of air from 54% to 19%. (**d**) Time-dependent integrated PL intensity of the nanofiber when relative humidity jumped from 33% to 54% (NF in the figure referred to the nanofiber, the whole Figure 17 was reprinted with permission from Meng et al. [42]).

One-dimensional waveguiding micro/nanofibers have shown great potential for optical sensing, with the high sensitivity and fast response due to their tight optical confinement [68]. Tong et al. constructed a humidity sensor using a semiconductor quantum dot (QD) doped polymer nanofiber, which showed excellent reversibility and long-term stability [42]. They suspended the polymer nanofiber across a microchannel, and all this was sealed inside a glass chamber. Optical launching and signal collection were completed through the two ends, which were placed outside of the nanofiber, as shown in Figure 17a. The surrounding relative humidity (RH) in the glass chamber was changed and the light outcoupled from the right end of the nanofiber when light was excited from the left end was recorded. Figure 17b demonstrates the varying change in PL intensity with RH; the reason was the passivation of surface trap states of QDs induced by water. The limit of detection was about 1% RH. Also, the sensor showed excellent reversibility on alternately cycling at 19% and 54% RH of air (Figure 17c), with response time less than 90 ms (Figure 17d), which is two orders of magnitude faster than the current RH sensors. Moreover, the above sensor system based on a polymer optical nanofiber was also suitable for detection of other sensitive substances, such as CN^–^; therefore, the polymer nanofiber sensor has a wide range of applications. 

## 3. Conclusions and Outlook

Recent progress on the polymer micro/nanofiber fluorescence waveguide has been briefly reviewed and summarized. Polymer optical micro/nanofibers represent an important class of photonic building blocks that exhibit fascinating optical functionalities. The researchers have conducted in-depth studies on their design, synthesis, modulation, and application and made impressive progress. The related optical devices have penetrated into various fields of photonics, resulting in a large number of new photonic components, such as optical modulators, optical sensors, and so on. In addition to the growth and corresponding applications, it is urgent to improve the various properties of the polymer micro/nanofiber fluorescence waveguide and integrate polymer optical micro/nanofibers to fabricate high-integrated optical components and circuits. Two main development directions are (a) the smart modulation of waveguide parameters during light propagation, and (b) the construction of different kinds of multifunctional heterojunctions, which are used to obtain different types of optical functional photonic components. The following are descriptions of these two directions.

**Figure 18:**
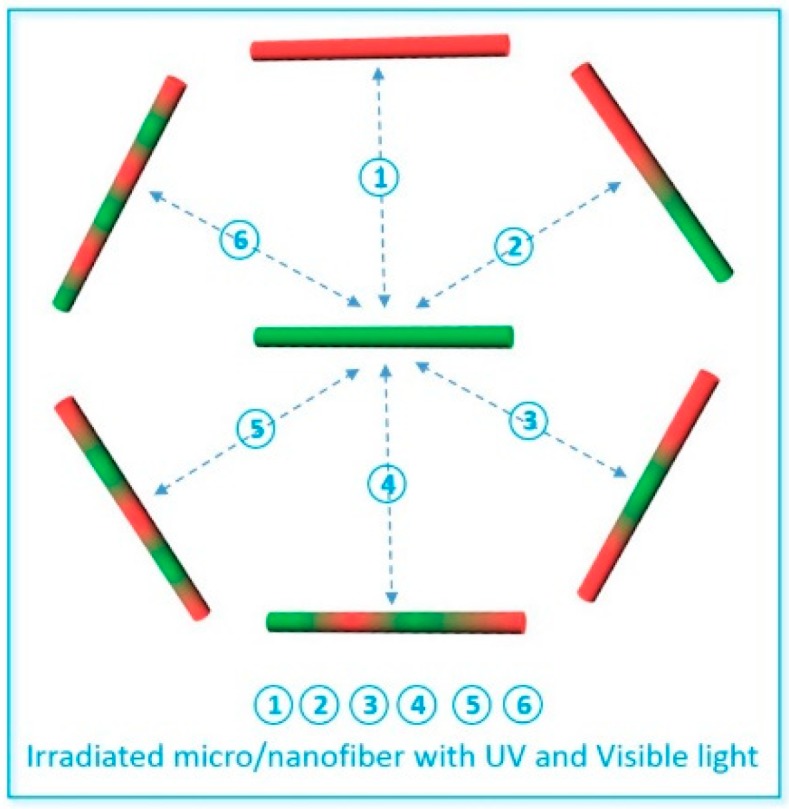
Schematic illustration of regulating the submicrostructure of a polymer micro/nanofiber. (1, 2, 3, 4, 5, 6 are the six examples that the micro/nanofiber was irradiated reversibly with UV and visible light of different region along the micro/nanofiber).

The micro/nanoscale polymer microstructure became an effective way to regulate photons, because the wavelength of photons is at the micro/nano scale. Combining the basic principles of photonics, designing and preparing the microstructure of polymer micro/nanofibers and integrating them to modulate photonic properties is an important research field in polymer micro/nanofiber photonics. A design of the modulation process combined with the photochrome within the micro/nanofiber is shown in Figure 18. By changing the composition and structure of the micro/nanofiber, effective control of photon generation, transmission, regulation, and detection can be achieved. When using UV light to irradiate the micro/nanofiber locally, photochromes in the irradiated regions undergo photoisomerization reactions that absorb the fluorescence of micro/nanofiber (from the conjugated polymer or the dye molecules) and emit their own fluorescence, while the nonirradiated regions emit the original fluorescence. The photochrome returns to the original state under visible light, and the structure and light emission of the micro/nanofiber also return to the same state as before light irradiation. Repeating the above process using UV and visible light to irradiate different regions of the micro/nanofiber as required and modulating the fluorescence of the same micro/nanofiber will yield different segmented structures. The effects of different irradiation light in different regions on the structure and luminescence of the polymer micro/nanofiber functionalized with photochromes can be studied.

**Figure 19:**
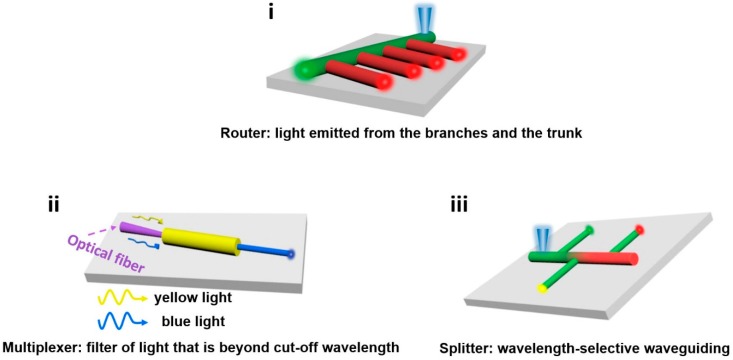
Schematic illustration of construction and performance study for optical components. (**i**) Multichannel router; (**ii**) wavelength division multiplexer; (**iii**) beam splitter.

Polymer micro/nanofibers with excellent optical properties can also be arranged and assembled into various types of heterojunctions, which could be employed as effective photonic components, where photons can be well confined, transmitted, and amplified. By studying the interaction and regulation mechanisms of these microstructures with light, different types of optical functional devices can be obtained, with the construction of photonics components as one of the final important goals. All of these provide a foundation and approaches for the development of new polymer micro/nanoscale optical waveguides and various types of photonic devices that have significance for the construction of integrated photonic circuits and optical chips. As shown in Figure 19, combining polymer micro/nanofibers with different luminosity and structures in composite structures by micromanipulation under a microscope with a long working distance objective lens, the optical signal in the waveguide micro/nanofibers can be coupled to the adjacent region through the evanescent field. By studying the synergistic effects between polymer micro/nanofibers with different structures and exploring the optical functions of the composite structures, we can construct different kinds of photonic components based on these multifunctional heterojunctions.

1. Construct a multichannel router. Dispose the photochrome functionalized color-changing micro/nanofiber and various luminous micro/nanofibers into a router structure, as shown in Figure 19a, and study how the mainline micro/nanofibers’ emissions affect the branch micro/nanofibers’ optical waveguides. 

2. Construct a wavelength division multiplexer. The propagation of optical signals is size-dependent, the optical waveguide function of the polymer micro/nanofiber is closely related to its cross-sectional size, and there is a cutoff dimension for light transmission, which is also called a short-pass filtering effect. To study the relationship between the transmission of light with different wavelengths in nanofibers and their diameters, combine a blue-emitting nanofiber with a diameter of approximately 800 nm and a yellow-fluorescent nanofiber with a diameter of approximately 400 nm, input the blue light and yellow light into the larger-diameter nanofiber on the left side at the same time, and record the emission waveguide from the right nanofiber end with smaller diameter (Figure 19b).

3. Construct a wavelength beam splitter. Combine micro/nanofibers with gradient-changing color and micro/nanofibers with different luminescence into a tree-like structure (Figure 19c). Excite the tip of the “tree trunk” site and collect the fluorescence emitted from the micro/nanofiber end of the “tree branch” in order to study the influence of different luminescence in different regions of the micro/nanofiber in the “tree trunk” on the optical waveguides of micro/nanofibers in the “tree branch.”
